# List of Diseases in London

**Published:** 1814-06

**Authors:** 


According to the report of several *Apothecaries* residing in various districts of the metropolis, the following was the average of the diseases in London between March 20th and April 19th:—

| | | | | | |
|---|---|---|---|---|---|
| Anasarca | 27 | Enterodynia | 8 | Peritonitis | 11 |
| Ascites | 11 | Enuresis | 3 | Phlegmasia Dolens | 1 |
| Asthma | 119 | Epilepsia | 12 | Phrenitis | 4 |
| Acne | 2 | Febris remittens | 149 | Pneumonia | 99 |
| Apoplexia | 16 | —— intermittens | 36 | Pleuritis | 2 |
| Aphtha | 21 | —— puerpera | 6 | Pleurodyne | 4 |
| Amenorrhœa | 28 | Gastritis | 3 | Polydipsia | 1 |
| Asthenia | 63 | Gastrodynia | 55 | Podagra | 36 |
| Abortio | 19 | Hæmatemesis | 7 | Porrigo larvalis | 14 |
| Bronchitis acuta | 16 | Hæmoptoe | 22 | —— scutulata | 8 |
| —— chronica | 22 | Hæmorrhois | 32 | —— favosa | 2 |
| Cancer | 2 | Hepatitis | 41 | Prurigo | 4 |
| Chlorosis | 17 | Hysteria | 34 | Psoriasis | 18 |
| Cholera | 28 | Hysteritis | 1 | Pyrosis | 15 |
| Chorea | 1 | Hydrocephalus | 11 | Rachitis | 4 |
| Cephalalgia | 73 | Hydrothorax | 7 | Rheumatis. acut. | 86 |
| Colica | 36 | Herpes | 44 | —— chron. | 97 |
| Catarrhus | 393 | Hypochondriasis | 12 | Roseola | 2 |
| Convulsio | 28 | Icterus | 15 | Rubeola | 69 |
| Coryza Maligna | 1 | Impetigo | 28 | Scabies | 81 |
| Carditis | 5 | Ischuria | 2 | Scarlatina simplex | 31 |
| Cynanche Tonsillaris | 112 | Leucorrhœa | 28 | —— anginosa | 22 |
| —— maligna | 11 | Lepra | 2 | —— maligna | 4 |
| —— Trachealis | 9 | Lichen | 13 | Splenitis | 2 |
| —— parotidea | 29 | Lumbago | 1 | Scrofula | 42 |
| Delirium Tremens | 3 | Mania | 9 | Synochus | 15 |
| Diabetes | 5 | Menorrhagia | 43 | Tabes Mesenterica | 12 |
| Dysenteria | 22 | Miliaria | 1 | Trismus | 1 |
| Diarrhœa | 132 | Morbi Infantiles | 273 | Typhus | 15 |
| Dyspnœa | 29 | —— Biliosi | 198 | Variola | 8 |
| Dyspepsia | 181 | Morbus Pediculosus | 1 | Varicella | 30 |
| Dysuria | 9 | Nephritis | 7 | Vermes | 47 |
| Eczema | 7 | Obstipatio | 58 | Vertigo | 29 |
| Ecthyma | 3 | Ophthalmia | 82 | Urticaria | 17 |
| Erythema | 6 | Paralysis | 14 | | |
| Erysipelas | 50 | Phthisis Pulmonalis | 60 | Total | 3792 |
| Enteritis | 17 | Pertussis | 89 | | |

The Deaths in the Bills of Mortality from March 15th to April 12th, 1814, according to the returns of the *Parish Clerks*, were as under:—

| | |
|---|---|
| Males | 840 |
| Females | 739 |
| Total | 1579 |

LONDON

